# *RUNX1* and *RUNX3* Genes Expression Level in Adult Acute Lymphoblastic Leukemia—A Case Control Study

**DOI:** 10.3390/cimb44080238

**Published:** 2022-08-01

**Authors:** Dagmara Szmajda-Krygier, Adrian Krygier, Krzysztof Jamroziak, Anna Korycka-Wołowiec, Marta Żebrowska-Nawrocka, Ewa Balcerczak

**Affiliations:** 1 Laboratory of Molecular Diagnostics and Pharmacogenomics, Department of Pharmaceutical Biochemistry and Molecular Diagnostics, Medical University of Lodz, Muszynskiego 1 Street, 90-151 Lodz, Poland; adrian.krygier@umed.lodz.pl (A.K.); marta.zebrowska@umed.lodz.pl (M.Ż.-N.); ewa.balcerczak@umed.lodz.pl (E.B.); 2 Department of Hematology, Institute of Hematology and Transfusion Medicine, Chocimska 5 Street, 00-791 Warsaw, Poland; krzysztof.jamroziak@interia.pl; 3 Department of Hematology, Medical University of Lodz, Ciolkowskiego 2 Street, 93-510 Lodz, Poland; anna.korycka-wolowiec@umed.lodz.pl

**Keywords:** adult leukemia, ALL, gene expression level, *RUNX* family genes, qPCR

## Abstract

The genetic factors of adult acute lymphoblastic leukemia (ALL) development are only partially understood. The Runt-Related Transcription Factor (*RUNX*) gene family play a crucial role in hematological malignancies, serving both a tumor suppressor and promoter function. The aim of this study was the assessment of relative *RUNX1* and *RUNX3* genes expression level among adult ALL cases and a geographically and ethnically matched control group. The relative *RUNX1* and *RUNX3* genes expression level was assessed by qPCR. The investigated group comprised 60 adult patients newly diagnosed with ALL. The obtained results were compared with a group of 40 healthy individuals, as well as clinical and hematological parameters of patients, and submitted for statistical analysis. ALL patients tend to have significantly higher *RUNX1* gene expression level compared with controls. This observation is also true for risk group stratification where high-risk (HR) patients presented higher levels of *RUNX1*. A higher *RUNX1* transcript level correlates with greater leukocytosis while *RUNX3* expression is reduced in Philadelphia chromosome bearers. The conducted study sustains the hypothesis that both a reduction and increase in the transcript level of *RUNX* family genes may be involved in leukemia pathogenesis, although their interaction is complex. In this context, overexpression of the *RUNX1* gene in adult ALL cases in particular seems interesting. Obtained results should be interpreted with caution. Further analysis in this research field is needed.

## 1. Introduction

Acute lymphoblastic leukemia (ALL) is a fatal disease with malignant transformation and proliferation of lymphoid precursor cells in the bone marrow, peripheral blood, and extramedullary sites [1]. Childhood ALL cases are successfully curable in almost 80%; however, among adults the indicators are not so satisfactory: while about 85% of patients achieve first remission, it is estimated that half of them will have an early relapse of leukemia [1,2]. There are also differences in the responses to the treatment depending on the age of the patient. Hence the need for distinguishing age groups and individual treatment regimen adjustment, which will allow older patients to achieve better results and effectiveness of therapy [2].

Molecular model of ALL development is only partially understood and characterized, further studies of genetic and epigenetic pathways are needed to understand the full mechanism of this type of leukemia progress. In the future it will allow to enhance diagnostics methods and develop new targets for personalized therapy, especially in older patients. Therefore, it appears necessary to investigate the genes engaged in hematopoiesis, particularly transcription factors that control or regulate other genes. Numerous studies support the contribution role of the Runt-Related Transcription Factor (*RUNX*) gene family members in hematological malignancies [3,4].

The Runt-Related Transcription Factor 1 (*RUNX1*) gene, located on chromosome 21, plays a significant role in hematopoiesis during embryonic development [5,6,7]. Its encoded protein acts as a transcription factor and forms a complex with the cofactor Core-Binding Factor Subunit Beta (CBFB) [5] which is responsible for ensuring the stability of the RUNX1 protein involved in the formation of hematopoietic stem cells and their differentiation into myeloid and lymphoid lines [3,5]. It is also necessary for CD8 T-cell development during thymopoiesis [5,8]. The loss of *RUNX1* function disturbs the differentiation of myeloid and lymphoid lines, which often leads to the development of leukemia [5,9]. The RUNX1 transcription factor is considered to be a regulator which, by mutation, can cause tumor-related epigenetic aberrations. Mutations are also found in a variety of cancers: loss of function mutations in T-cell acute lymphoblastic leukemia suggests a role for RUNX1 as a suppressor in T-cell transformation; somatic mutations were also detected in breast cancer [4,8]. *RUNX1* expression is reduced in solid tumors prone to metastasis, indicating its role in suppressing tumor progression [4,7]. However, increased expression of *RUNX1* can also be oncogenic: amplification of the chromosome region 21q that contains the *RUNX1* gene (for example, chromosome trisomy 21 in Down syndrome) has been observed in ALL from the B lineage precursors and AML; high levels of *RUNX1* expression have also been found in various epithelial neoplasms (including skin cancer, endometrioid tumor) [4].

The Runt-Related Transcription Factor 3 (*RUNX3*) gene, located on chromosome 1 also encodes transcription factor and is strongly expressed in all hematopoietic stem cells [10,11,12]. It is essential in the process of thymopoiesis, T-cell differentiation, and the proper expression of CD4 and CD8 antigens [7,8]. However, its biological function is largely unexplored. The *RUNX3* gene is noted to be involved in oncological events [7]. There is evidence that inactivation of this gene is associated with the development of various cancers (for example breast cancer, gastric cancer) [13,14,15]. Nonetheless, *RUNX3* overexpression is frequently observed in head and neck and in ovarian cancer [16,17]. The *RUNX3* gene has been described as both a tumor suppressor and promoter, sometimes with contradicting results in the same type of tumor, which most likely reflects the complex role of this gene and its encoded factor in oncogenesis [7,12]. In this context, changes in the level of gene expression seem to be particularly important [7].

Therefore, the purpose of this study was the assessment of relative *RUNX1* and *RUNX3* genes expression level among adult acute lymphoblastic leukemia cases, the results were compared with the control group and connected with some important clinic-pathological features in order to elucidate the role of selected genes in ALL.

## 2. Materials and Methods

### 2.1. Materials

Sixty patients (38 women, 22 men, mean age 58 years) newly diagnosed with acute lymphoblastic leukemia were enrolled in the study. The diagnosis and risk stratification were established at the Institute of Hematology and Transfusion Medicine in Warsaw and Department of Hematology, Medical University of Lodz (Poland), according to the World Health Organization (WHO) 2016 criteria. The control group included 40 voluntary blood donors, ethnically and geographically matched to the investigated group. RNA isolated from peripheral blood samples of subjects comprised the research material. Investigation was conducted in accordance with the Declaration of Helsinki principles and was approved by the Medical University of Lodz Ethics Committee (RNN/88/16/KE, RNN/285/13/KE along with the KE/227/20 amendment). Informed consent was obtained from all individuals prior to their participation in the study. All data were collected and stored anonymously.

### 2.2. Sample Preparation and Experiment Conduction

RNA extraction was performed using a Total RNA Mini kit (A&A Biotechnology, Gdynia, Poland), according to the manufacturer’s guidelines. The quality of the obtained RNA samples was assessed spectrophotometrically using an absorbance ratio of 260 nm to 280 nm (A260/A280—DNA/RNA absorbance to protein absorbance). In all samples, the ratio ranged from 1.8 to 2.0, which proves the good quality of the extracted material. Absorbance at 260 nm was used to establish the amount of RNA required for the reverse transcription reaction. Further analyses were then implemented using cDNA acquired from the same amount of input RNA. Final concertation of RNA in investigated cases was set at 0.01 μg/μL.

Reverse transcription reaction in order to obtain cDNA was performed using a High-Capacity cDNA Reverse Transcription kit (Applied Biosystems; Thermo Fisher Scientific, Inc., Waltham, MA, USA), following the manufacturer’s protocol, with thermal cycling parameters set at: 25 °C for 10 min, then at 37 °C for 120 min, finally at 85 °C for 5 min.

In order to assess the expression level of selected genes quantitatively, the qPCR technique was performed using a Rotor-Gene™ 6000 thermocycler (Corbett Life Science; Qiagen GmbH, Hilden, Germany). To acquire the threshold cycle and the relative expression level of the selected genes, Glyceraldehyde-3-Phosphate Dehydrogenase (*GAPDH*), a housekeeping gene, was chosen as an endogenous reference. The primers sequences were as follows: *RUNX1* F 5′-AGTGGAAGAGGGAAAAGC-3′, R 5′-ATCCACTGTGATTTTGATGG-3′; *RUNX3* F 5′-ATGACGAGAACTACTCCG-3′, R 5′-TCAGGGTGAAACTCTTCC-3′. Reaction mixture contained: 0.7 μL of 10 μM each primer, 5 μL of RT HS-PCR Mix Sybr^®^ B (A&A Biotechnology, Gdynia, Poland), and 1 μL of cDNA template. Nuclease- and RNase-free water was then added to the total volume of 10 μL. Thermal cycling conditions were set as follows: initial activation for 10 min at 95 °C, followed by 40 cycles of denaturation for 10 s at 95 °C, primer annealing for 15 s at 55 °C for *RUNX1,* and 58 °C for *RUNX3*, followed by elongation for 20 s at 72 °C. The analysis was conducted in triplicates for each sample and carried out in separate tubes for reference and investigated genes. To ensure the quality of the assay in every trial no template control (NTC) samples were included. A post-amplification melting-curve analysis was performed in order to check the reaction specificity (Figure S1). The obtained Ct values were averaged. In order to estimate relative changes in the expression level, the 2^−ΔΔCq^ method was selected [18].

The STATISTICA 13.1 (StatSoft Inc., TIBCO Software Inc., Palo Alto, CA, USA) software package was used for statistical analyses. The Shapiro–Wilk test was used for verification of numerical data with normal distribution. Variables with normal distribution were compared between subgroups using the Student *t*-test. Variables with nonparametric distribution were compared using the Mann–Whitney U test. The association between two quantitative traits was evaluated by either Spearman’s correlation coefficient or Pearson’s r correlation; a *p*-value less than 0.05 was considered a statistically significant difference.

## 3. Results

Relative expression level of *RUNX1* and *RUNX3* genes was highly variable among the selected ALL cases and it ranged between 0.13 and 7.17 for the *RUNX1* gene and between 0.06 and 44.77 for the *RUNX3* gene. Patients (38 women, 22 men) were divided into two groups according to ALL subtype to B-ALL (47 cases) and T-ALL (13 cases). The groups’ mean age of leukemia onset was 58 years, 59 years for women and 56.5 years for men. Detailed demographic and clinical characteristics of patients are shown in Table 1. Relative expression level of selected genes was further compared with clinic-pathological features.

### 3.1. Comparison of RUNX1 and RUNX3 Expression Level between Investigated and Control Group

**Figure 1:**
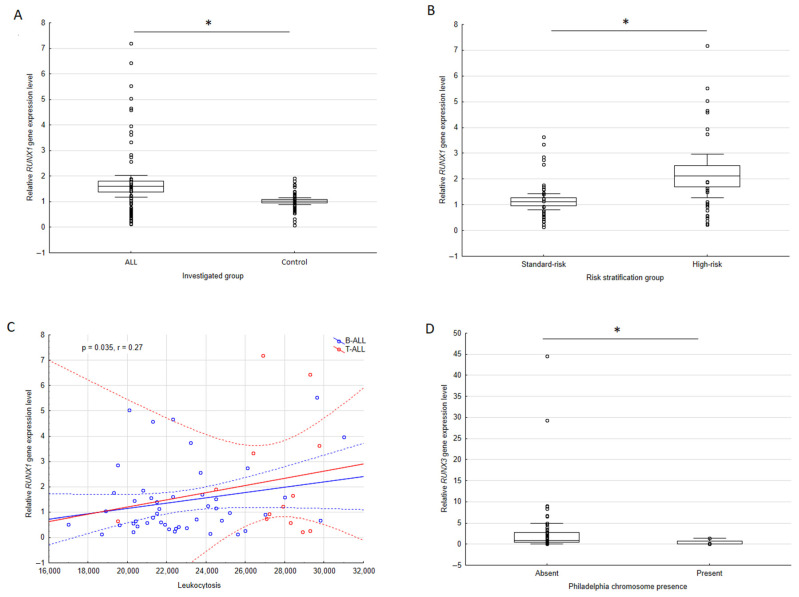
(**A**)—box-whiskers plot for *RUNX1* gene expression level comparison between ALL and control group, data presented as mean, standard error, and 95% confidence interval, raw data are presented as individual dots; (**B**)—box-whiskers plot representing the difference in *RUNX1* gene expression level between the high- and standard-risk patients, data presented as mean, standard error and 95% confidence interval, raw data are presented as individual dots; (**C**)—scatterplot showing correlation between indicated *RUNX1* gene expression and leukocytosis of ALL patients with subdivision for B-ALL (blue) and T-ALL (red), line represents a linear model fit whereas the dashed line indicates 95% confidence interval, each dot represents an individual; (**D**)—box-whiskers plot representing the differences in *RUNX3* gene expression and Philadelphia chromosome presence, data presented as median, interquartile and non-outlier range, raw data are presented as individual dots. * *p* < 0.05.

In the first step of analysis the *RUNX1* and *RUNX3* genes expression level between the investigated and control group was compared. The analysis revealed that the *RUNX1* gene expression levels were significantly higher in ALL patients in comparison to controls (*p* = 0.028, Figure 1A). The results of *RUNX3* levels did not differ between the two groups (*p* = 0.123).

### 3.2. RUNX1 Relative Expression Level Statistical Analysis

Comparison of patients’ leukemia subtype and the relative *RUNX1* expression level showed no statistically significant differences (*p* = 0.302). The analysis performed for risk group stratification revealed that high-risk (HR) patients presented higher levels of *RUNX1* compared with standard-risk (SR) individuals (*p* = 0.012, Figure 1B). Then, the relative expression of *RUNX1* mRNA, depending on gender among the investigated group, was rated. No meaningful difference was obtained, although the *p*-value was near the significance border (*p* = 0.064). The levels were slightly higher in men.

Another compared parameter was the age of leukemia onset in the patients. No significant correlations were revealed between the group age of leukemia onset and relative *RUNX1* expression level (*p* = 0.575). Association between *RUNX1* expression level, leukocytosis, and blast percentage in the bone marrow was evaluated subsequently. Statistical analysis indicated a significant positive correlation between *RUNX1* mRNA level and leukocytosis (*p* = 0.035, r = 0.27), higher expression levels were found in patients with greater leukocytosis (Figure 1C). However, the investigation revealed no meaningful correlation between the *RUNX1* gene and blast percentage (*p* = 0.771). Additionally, the relative *RUNX1* expression level did not significantly differ among Ph carriers and non-carriers (*p* = 0.380). Amongst the B-ALL patients the presence of CD10 marker was additionally evaluated, analysis revealed no significant differences in the *RUNX1* mRNA level between CD10-positive and CD10-negative trials (*p* = 0.987).

### 3.3. RUNX3 Relative Expression Level Statistical Analysis

In the next part of the study, dependences of the relative expression level of *RUNX3* with clinic-pathological parameters were evaluated. Relative *RUNX3* level did not significantly differ in patients with B-ALL and T-ALL subtypes (*p* = 0.670). Analysis of risk stratification groups showed no differences in the *RUNX3* transcript level between the HR and SR patients (*p* = 0.557). Furthermore, the mRNA expression of this gene does not depend significantly on gender (*p* = 0.642). The search for the association between age of leukemia onset, leukocytosis, and the blasts content in the bone marrow with the relative *RUNX3* expression did not provide statistically significant results (*p* = 0.954, *p* = 0.910, *p* = 0.810, respectively). A statistically meaningful difference was found between *RUNX3* expression and the Philadelphia chromosome presence (*p* = 0.023), Ph carriers tended to have lower relative *RUNX3* levels (Figure 1D). Amongst the B-ALL patients, the analysis revealed no significant differences in *RUNX3* mRNA level between CD10-positive and CD10-negative trials (*p* = 0.599).

### 3.4. Comparison of RUNX1 and RUNX3 Expression Level between ALL and AML Patients

Moreover, in one of our earlier studies the *RUNX1* and *RUNX3* expression level assessment was performed on a group of patients diagnosed with acute myeloid leukemia (AML) [19]. Therefore, the difference in *RUNX1* and *RUNX3* expression level was evaluated between the investigated ALL cohort (*n* = 60) and 43 adult AML cases, geographically and ethnically matched to the study group. Statistical analysis revealed no differences in the *RUNX1* and *RUNX3* gene transcript level according to acute leukemia type (*p* = 0.469, *p* = 0.539, respectively).

## 4. Discussion

The course of lymphoid differentiation is firmly controlled by hierarchical activation of transcription factors and selection via functional signal transduction. Acute lymphoblastic leukemia represents a group of lymphoid neoplasms in the B or T precursor stage, arising from genetic modifications that inhibit lymphoid maturation and lead to abnormal cell proliferation and survival. The clinical heterogeneity in the course and outcome of this leukemia, particularly when comparing pediatric populations with adult ALL cases, reflects the possibility of diverse biological subtypes of this malignancy [20].

Until recently there has been very little information about the investigated genes expression level in adult acute lymphoblastic leukemia cases. *RUNX* family members are responsible for hematopoietic stem cells differentiation into myeloid and lymphoid lines. Taniuchi et al. found that *RUNX1* is crucial for active repression in CD4-negative/CD8-negative thymocytes, while *RUNX3* is essential to establish epigenetic silencing in cytotoxic lineage thymocytes [8]. *RUNX1* mutations were previously shown to be associated with acute myeloid leukemia occurrence [21,22] and were also found in ALL [23,24,25]. Studies carried out by other research groups indicate an important link between *RUNX1* gene and leukemogenic factors, e.g., by affecting the Myb and Myc enhancers [26]; moreover, on the correlation between *RUNX1* and *RUNX3* genes and *NOTCH1* pathway [27]. The study undertaken by Goyama et al. indicated a significant link between the function of the selected genes and leukemic cell survival [28]. The *RUNX1* gene is frequently deregulated in childhood B-cell precursor acute lymphoblastic leukemia (BCP-ALL), where the *ETV6-RUNX1* translocation causes an overexpression of the *RUNX1* gene [29,30,31], on the other hand this gene can also be aberrantly expressed in T-ALL cases, where it is reported to be oncogenic in the T-cell lineage and to cause lymphoma in mice [32]. Despite the similar disease symptoms to childhood leukemia, the adult ALL seems to be a different entity with an altered genetic background and the specific expression pattern is still unclear.

Since all these data suggest that RUNX family members are crucial for normal hematopoiesis and change in their structure or expression can cause leukemia development, the aim of this study was to evaluate *RUNX1* and *RUNX3* relative expression level in adult acute lymphoblastic leukemia cases.

Currently, it has been hypothesized that, due to the different expression level, the *RUNX1* gene may play an oncogenic or suppressor role depending on the type of malignant tumor or hematological neoplasms [7,33]. Fu et al. reported that in cytogenetically normal acute myeloid leukemia (CN-AML), a higher level of *RUNX1* expression is associated with poorer prognosis, patients with high expression values showed a shorter overall survival (OS) and a shorter symptom-free survival (EFS) [33]. Moreover, Wilkinson et al. found that high *RUNX1* expression is associated with a detectable level of MRD on the 29th day of therapy in ALL patients with MLL fusion genes, and thus with a poorer clinical course and risk of early relapse [34]. The present study revealed that the group of ALL patients had significantly higher *RUNX1* gene expression level compared with the controls (*p* = 0.028); furthermore, the high-risk group of ALL patients also presented significantly higher *RUNX1* levels (*p* = 0.012). These results may indicate that *RUNX1* acts as an oncogene in the adult ALL process. Moreover, it was found that high levels of *RUNX1* gene expression correlate with higher leukocytosis in the investigated ALL cohort (*p* = 0.035). High leukocytosis is a recognized negative prognostic factor in ALL patients, which may imply a more aggressive disease course. In this study, the level of *RUNX1* mRNA gene is not dependent on the sex and age of leukemia onset in patients, similar results in AML were obtained by a team led by Fu et al. [33] and Krygier et al. [19]. There are several factors that may have led to the altered *RUNX1* level of expression in ALL patients. This commonly disrupted gene in hematological malignancies has a well-characterized molecular consequence in less than half of the cases [31]. The *RUNX1* gene is frequently targeted by translocations or mutations which cause mostly loss of its function, but the observed molecular changes can also aberrantly upregulate *RUNX1*. One of them, the t(12;21)(p13;q22)/*ETV6-RUNX1* found in B-lineage acute lymphoblastic leukemia, can be mentioned. It was suggested that this disruption might deregulate the *RUNX1* promoters and cause *RUNX1* overexpression in *ETV6-RUNX1*-positive patients without concurrent *RUNX1* gain [30,31]. Other novel translocation involving *RUNX1* aberrant upregulation is t(5;21)(q13;q22), which was acquired during the progression of myelodysplastic syndrome to AML in a pediatric patient [31]. Additional factors important in regulating *RUNX1* transcription level are gene regulatory elements (REs), such as enhancers located in non-coding DNA regions. A recent bioinformatics study identified recurrent mutations in REs that affect gene function, including *RUNX1* and are connected to hematological malignancies development [35]. Furthermore, aberrant splicing and splicing factor mutations are suggested to contribute to the mechanism of *RUNX1* upregulation [36]. What is more, epigenetic mechanisms regulating genes expression also seem to influence *RUNX1*, the overexpression motif of RUNX1 due to demethylation in various regions (DNA-binding sites, including promoter region) was shown in the HEK−293T-cell line and also in human peripheral blood CD34+ hematopoietic progenitor cells and CD14+ monocytes [37].

To date, expression levels of the *RUNX3* gene have been assessed in a wide variety of neoplastic diseases, including AML. Krygier et al. stated that the *RUNX3* gene may serve as a prognostic factor in acute myeloid leukemia because its higher expression levels were associated with higher mortality among the study group. This seems to confirm the oncogenic nature of this gene in AML [19]. Cheng et al. and Lacayo et al. reported similar observations among AML patients [38,39], where pediatric patients with high expression values had shorter OS and EFS rates. In contrast, Haider et al. presented evidence of the *RUNX3* gene suppressive nature in human T-cell tumors, particularly in patients suffering from Sézary’s syndrome, where ectopic RUNX3 secretion, as a result of increased gene expression levels, reduces viability and induces apoptosis of neoplastic cells [10]. Thus, it may seem that *RUNX3* acts differently in various malignancies and both an increase and reduction in gene expression level may contribute to the development and progression of neoplastic disease. In this study, no differences in the *RUNX3* expression levels between investigated and control groups were observed. However, the reduced *RUNX3* levels correlated with Ph chromosome occurrence in adult acute lymphoblastic leukemia cases (*p* = 0.023). The Ph chromosome appears to negatively regulate the *RUNX3* gene transcript level, though there is a large disproportion in size between the two subgroups and no final conclusion can be made. Information about *RUNX3* gene expression level in adult ALL with *BCR-ABL* fusion is limited. The available scientific sources only state that potential downregulation of *RUNX3* expression is caused by hypermethylation of P1 and/or P2 promoters. This was reported in AML and chronic myeloid leukemia (CML), as well as gastric cancer cells, suggesting that the hypermethylation of *RUNX3* reduces its expression during cancer development. Furthermore, it was proposed that the weak expression of the *RUNX3* gene might collaborate with a preferential genetic mutation, such as *BCR-ABL1* [40]. Other reports stated that the high expression of the *RUNX3* gene and protein level in mice and in cell lines with the present *Bcr-abl* fusion resulted in resistance to treatment with imatinib and this effect was lost in mutant *RUNX3* [41,42]. Unfortunately, to the best of our knowledge, there are no other reports as to the possible functional influence of these genes on each other, especially in the material obtained from patients, hence the discussion is limited. Cheng et al., as well as Krygier et al. described the lack of correlation between the observed *RUNX3* gene expression level and demographic and clinical data among AML patients, such as: gender, age at diagnosis, and FAB classification [19,38]. The results obtained in this study are similar since no association was identified between gender, age of leukemia onset, ALL subtype, risk stratification, and the relative mRNA level of the *RUNX3* gene.

We are aware of the limitations of the study. Gene expression was analyzed in total peripheral blood samples, whose cellular compositions may be significantly different between controls and ALL samples. Thus, the observed alterations in *RUNX1* levels may be due to the differences in the proportion of each cell type. It must be mentioned that the obtained results are restricted to the Polish population with a limited sample size. In order to confirm the presented outcomes, further trials on larger groups of ALL patients from diverse populations are required. Lack of association between other selected features may be caused by a relatively small group of the investigated patients. Future trials would benefit from increasing the number of subjects, especially those with the T-ALL subtype. The conducted study sustains the hypothesis that both a reduction and increase in the transcript level of genes from the *RUNX* family may be involved in pathogenesis of leukemia, although their interaction is complex. In this context, overexpression of the *RUNX1* gene in adult ALL cases in particular seems interesting.

## Figures and Tables

**Table 1:** Demographic and clinical characteristics of patients.

Characteristics	Number of Patients (*n* = 60)
Gender	
Men	22
Women	38
Age of leukemia onset (years)	
Range (mean)	35–81 (58)
Leukemia subtype	
B-ALL	47
T-ALL	13
Risk stratification group	
Standard-risk (SR) High-risk (HR)	36 24
Leukocytosis (/μL)	
Range (mean)	17,000–31,000 (23,732)
Blast percentage	
Range (mean)	67–99 (83)
Philadelphia Chromosome	
Positive	4
Negative	56
CD10 marker (B-ALL)	
Positive	41
Negative	6

## Data Availability

The datasets used and/or analyzed during the current study are available from the corresponding author on reasonable request.

## Supplementary Materials

The following supporting information can be downloaded at: www.mdpi.com/article/10.3390/cimb44080238/s1, Figure S1: Melting curves of the studied genes; A, *RUNX1*; B, *RUNX3*.
