# Supplementary figures and images for: RUNX1 and RUNX3 Genes Expression Level in Adult Acute Lymphoblastic Leukemia—A Case Control Study
Source: Curr Issues Mol Biol. 2022 Aug 1;44(8):3455–64. doi: 10.3390/cimb44080238 (PMC9406551; doi:10.3390/cimb44080238)

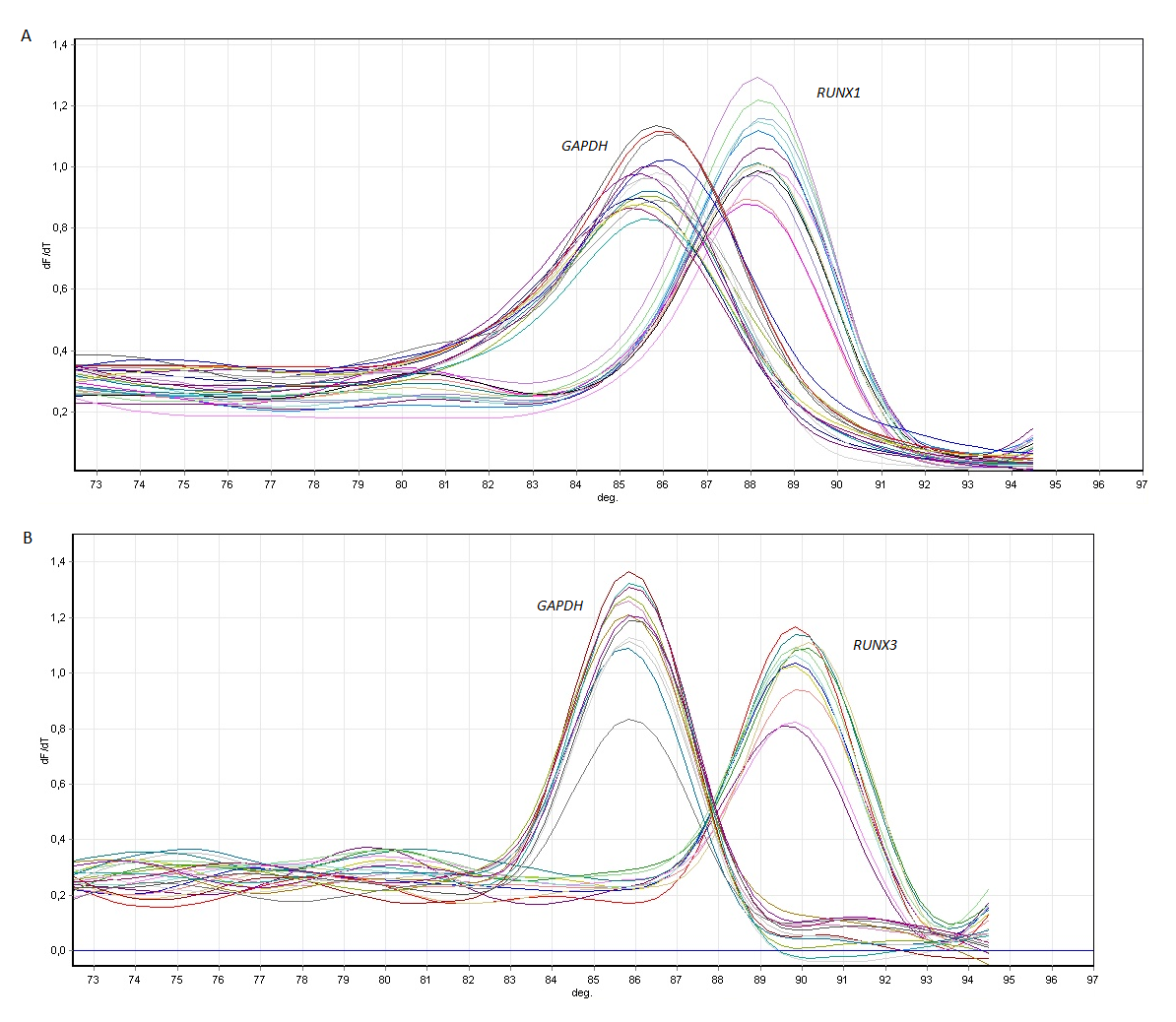

Supplement: Supplementary file 1 [file cimb-44-00238-s001.zip › Figure S1.tif]
